# Abdominal Surgery by Untrained Operators with Deficient Appliances

**Published:** 1893-09

**Authors:** 


### Editorial.

### ABDOMINAL SURGERY BY UNTRAINED OPERATORS WITH DEFICIENT APPLIANCES.

The discussion going on recently in regard to the recognition of emergency work in surgery, indicates that while the higher type of operative procedures should always have precedence, there is a place for that class of measures called for at times by the necessities of the case. Of course no one unqualified for surgical work or without the means of executing it properly, should undertake the management of a case, when a surgeon of experience can be secured to do it. But the question is raised, whether in the absence of an expert operator, any member of the profession who is called to see a patient suffering from recent traumatism should feel authorized to meet this exigency with promptness by a life saving operation, when delay would prove fatal.

If it were a case of hemorrhage from the division of an artery, though temporary compression would arrest the bleeding for the time, it could not avail for permanent relief, and ligation of the vessel should be resorted to, even by the most inexperienced practitioner, without the ordinary appliances for such an operation. But the class of cases which has elicited this discussion is of a different nature, and there may be degrees of urgency, in all of which interference is not imperative, and in regard to which it is questionable whether an untrained operator would be warranted in proceeding. This issue is made more especially in penetrating wounds of the abdomen and thorax. Should there be a protrusion of the abdominal viscera from an incised wound of the parietes, and it was found that the coats of the intestine were cut or that a mesenteric artery was divided, it is evident that there should be no hesitation on the part of the medical practitioner, least qualified for surgical work, about proceeding to suture the openings and ligate the vessel, without waiting for a specialist.

But in the event of perforations without protrusion, a grave question arises in the diagnosis and scarcely less grave in the results of cases left alone, and those which have undergone laparotomy. It is not generally taken into account that the facts as recorded show a larger mortality from the cases of abdominal injury which have been operated upon, than from those treated without operation. I drew attention to this matter in the discussion of Dr. Link's paper before the surgical section of the American Medical Association at Milwaukee and the inference may be drawn, that if the percentage of mortality has been increased by laparotomy in the hands of skilful operators, we should not expect better results from unskilful use of the knife and the needle.

It would seem, therefore, unwise to encourage the laying open of the abdomen by medical men without surgical experience in this class of cases. It is preferable to leave a patient with abdominal injury to be treated without operation, that may or may not suffer from visceral lesion, to having an operation performed without all due precautions in the steps taken from beginning to end. The great practical deduction to be made from a consideration of the subject is that abdominal surgery should receive the most careful attention of those who expect to undertake the treatment of visceral injuries, and in view of the mortality which has attended laparotomy in this class of cases it is not expedient that those without skill and experience should be encouraged to open the abdomen.

J. McF. G.

